# Characterization of Virulent West Nile Virus Kunjin Strain, Australia, 2011

**DOI:** 10.3201/eid1805.111720

**Published:** 2012-05

**Authors:** Melinda J. Frost, Jing Zhang, Judith H. Edmonds, Natalie A. Prow, Xingnian Gu, Rodney Davis, Christine Hornitzky, Kathleen E. Arzey, Deborah Finlaison, Paul Hick, Andrew Read, Jody Hobson-Peters, Fiona J. May, Stephen L. Doggett, John Haniotis, Richard C. Russell, Roy A. Hall, Alexander A. Khromykh, Peter D. Kirkland

**Affiliations:** Elizabeth Macarthur Agriculture Institute, Menangle, New South Wales, Australia (M.J. Frost, J. Zhang, X. Gu, R. Davis, C. Hornitzky, K.E. Arzey, D. Finlaison, P. Hick, A. Read, P.D. Kirkland);; The University of Queensland, St Lucia, Queensland, Australia (J.H. Edmonds, N.A Prow, J. Hobson-Peters, F.J. May, R.A. Hall, A. A. Khromykh);; University of Sydney and Westmead Hospital, Westmead, New South Wales, Australia (S.L. Doggett, J. Haniotis, R.C. Russell)

**Keywords:** Australia, Kunjin virus, virulence, West Nile virus, glycosylation, horses, encephalitis, viruses, arboviruses

## Abstract

To determine the cause of an unprecedented outbreak of encephalitis among horses in New South Wales, Australia, in 2011, we performed genomic sequencing of viruses isolated from affected horses and mosquitoes. Results showed that most of the cases were caused by a variant West Nile virus (WNV) strain, WNV_NSW2011_, that is most closely related to WNV Kunjin (WNV_KUN_), the indigenous WNV strain in Australia. Studies in mouse models for WNV pathogenesis showed that WNV_NSW2011_ is substantially more neuroinvasive than the prototype WNV_KUN_ strain. In WNV_NSW2011_, this apparent increase in virulence over that of the prototype strain correlated with at least 2 known markers of WNV virulence that are not found in WNV_KUN_. Additional studies are needed to determine the relationship of the WNV_NSW2011_ strain to currently and previously circulating WNV_KUN_ strains and to confirm the cause of the increased virulence of this emerging WNV strain.

## Abstract 2

An encephalitis outbreak among horses was caused by a pathogenic variant of Kunjin virus.

In Australia, Murray Valley encephalitis virus (MVEV) and West Nile virus (WNV) Kunjin (KUN) strain are the main etiologic agents of arboviral encephalitis in humans, which usually occurs as isolated sporadic cases or occasional small outbreaks, mainly in northwestern Australia and rarely in southern regions (Figure 1, panel A) (*1*). MVEV is the more virulent pathogen and the only proven cause of fatal arboviral encephalitis in humans in Australia (*2*). WNV_KUN_ infections are infrequent and less severe (*3*). Horses are also susceptible to these viruses and have been involved in WNV outbreaks elsewhere, most notably in the United States in an outbreak that began in 1999. In Australia, infection with WNV_KUN_ has been detected intermittently in horses in the Southeast, but reports of encephalitis caused by this virus are rare (*3*). In New South Wales (NSW), Australia, the seroprevalence of WNV_KUN_ in horses is <5% (P.D. Kirkland and A. Read, unpub. data); infection is confined to inland areas where flooding supports large mosquito populations and water birds are a reservoir and amplifying host. Even in years when WNV_KUN_ has caused disease in humans, disease has rarely been observed or confirmed in horses (*3**,**4*).

**Figure 1:**
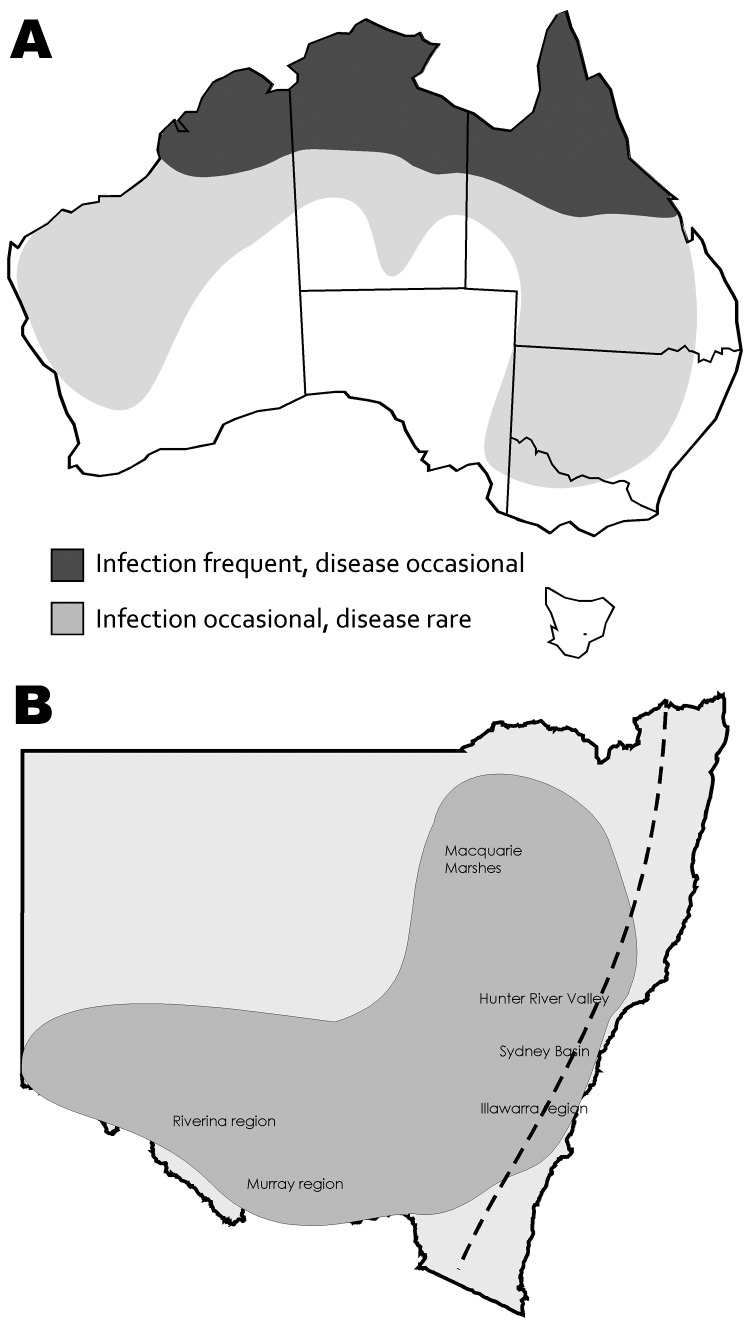
Known distribution of West Nile virus infection and disease caused by Kunjin strain (A) and distribution of encephalitis cases among equids (B), New South Wales, Australia, 2011. Dashed line indicates the Great Dividing Range.

In 2011, an outbreak of encephalitis occurred among horses in NSW. To analyze this strain of WNV_KUN_, we conducted genomic sequencing, antigenic profiling, in vitro growth kinetics, and mouse virulence studies on virus isolates from diseased animals and mosquitoes.

## Materials and Methods

### Disease Outbreak

In late February 2011, neurologic disease was reported in several horses in northwestern and southwestern NSW. The number of cases and geographic distribution gradually increased. By mid-June 2011, specimens from ≈300 horses were submitted to the virology laboratory at Elizabeth Macarthur Agriculture Institute (Menangle, NSW, Australia). Many more horses probably were affected. Diseased horses were located throughout most of NSW, west of the Great Dividing Range, but also extending through the Hunter River Valley region, Sydney Basin, and Illawarra coastal region immediately south of Sydney (Figure 1, panel B). Cases also occurred in other southern Australia states. Clinical signs were generally consistent with those described in horses infected with WNV in the United States (*5*). A detailed report of the clinical signs, virology, and pathology of equine cases will be published elsewhere.

### Specimen Collection

Whole brains were removed from 12 horses at postmortem examination. Half of each brain was fixed in 10% neutral buffered formalin; the other half was held fresh at 4°C. Upon receipt, we collected small pieces of fresh and formalin-fixed tissue from several locations in the cerebrum and cerebellum and along the brain stem and cervical spinal cord. If virus isolation could not be performed on fresh samples within 24 h after receipt, we held the samples at −80°C until tested. Before testing, we prepared 10% tissue homogenates in RPMI medium (Life Technologies, Carlsbad, CA, USA) containing antimicrobial drugs.

Mosquitoes were collected throughout NSW, as part of the NSW Arbovirus Surveillance and Mosquito Monitoring Program, by using dry ice–baited light traps. The mosquitoes were submitted live to the Medical Entomology Laboratory at Westmead Hospital (Westmead, NSW, Australia) for species identification, arbovirus isolation, and virus identification (*6*).

### Cells and Viruses

We propagated Vero 76 cells in Dulbecco modified minimum essential medium (DMEM; Life Technologies) supplemented with 10% fetal bovine serum (FBS). C6/36 *Aedes albopictus* mosquito cells were maintained in RPMI medium supplemented with 10% FBS, and BHK21 cells were maintained in DMEM containing 5% FBS. All cell lines were obtained from the American Type Culture Collection (Manassas, VA, USA). We used prototype WNV_KUN_ MRM61C (*7*) and WNV New York 99 (WNV_NY99_) 4132 strains (*8*) for comparison with WNV_NSW2011_. Stocks of WNV_KUN_ MRM61C and WNV_NY99_ 4132 that most closely resembled the low-passage level WNV_NSW2011_ were prepared by electroporation of BHK21 cells with WNV_KUN_ or WNV_NY99_ RNA (prepared from corresponding infectious cDNA clones) and passaging them 1× in C6/36 cells. Viral supernatants were harvested 5 days later. Viral titers for each viral stock were determined by plaque assay on Vero 76 cells.

### Virus Neutralization Tests

We conducted microneutralization tests (*9*) in Vero cells by using 25–100 infectious units (measured as 50% tissue culture infective doses) of WNV_NSW2011_, WNV_KUN_, and WNV_NY99_; we used 2-fold dilutions of serum from an initial dilution of 1:20. Results were scored as 80% reduction in virus growth or 100% inhibition of virus growth. Reduction in virus growth was determined by assessing the extent of cytopathic effect in each well. Inhibition of virus growth was determined by the absence of viral antigen in the cells of each well when tested with a WNV-reactive monoclonal antibody (mAb) in ELISA.

### Nucleic Acid Purification

We used the MagMax-96 Viral RNA Isolation Kit (Ambion, Austin, TX, USA) on a magnetic particle handling system (Kingfisher 96; Thermo Electron Corporation, Vantaa, Finland) to extract total nucleic acid from clarified 10% brain homogenate (50 μL) or tissue culture fluid. Purified nucleic acids were eluted in 50 μL of kit elution buffer and used immediately for PCR amplification or stored frozen at ≈−20°C

### Real-Time Reverse Transcription PCR

We used a published WNV real-time reverse transcription PCR (rRT-PCR) (*10* [assay 2]) with the following variations: Black Hole Quencher (Biosearch Technologies, Novato, CA, USA) was used instead of TAMRA on the probe, the internal control system was not used, and 5 μL of RNA was used as template. The AgPath-ID One-Step RT-PCR Kit (Applied Biosystems, Foster City, CA, USA) was used for the rRT-PCR on a 7500HT Fast Real-Time PCR System (Applied Biosystems). We ran rRT-PCR reactions in standard mode, according to conditions recommended by the mastermix manufacturer.

### Virus Isolation

Supernatant from the 10% brain homogenate was placed on monolayers of *A. albopictus* C6/36 mosquito cells in cell culture tubes. The cultures, which were maintained in RPMI medium containing antimicrobial drugs at standard concentrations and supplemented with 2% FBS, were incubated at 28°C for 5–7 days. Culture supernatants were then passaged up to 3× on BHK21 cells maintained in Basal Medium Eagle (MP Biomedicals, Sydney, Australia) containing antimicrobial drugs and 2% FBS. We regularly examined cultures by light microscopy for cytopathic effects. We used rRT-PCR to confirm the identity of virus isolates in culture supernatants or to confirm that there was no virus replication in the absence of cytopathology. Viral RNA recovered from culture fluid at the first or second passage in BHK21 cells was sequenced as described below. A virus isolate obtained from the first brain examined was designated WNV_NSW2011_. Virus isolation was conducted on homogenates of mosquitoes by similar methods but with passage onto BHK and PSEK cells after initially being placed on C6/36 cells. We identified virus isolates by using immunoassays with generic and specific mAbs.

WNV_NSW2011_ virus harvested from the first passage in C6/36 cells was used to examine plaque morphology and virulence in mice. The virus was passaged 1× in Vero76 cells for 4 d and 1× in C6/36 cells for 5 d. Virus supernatant was centrifuged at 500 × *g* at 4°C for 5 min before being stored at −80°C

### Reactivity with mAbs

Reactivity of the new isolate with a panel of mAbs was compared with that of WNV_KUN_ and WNV_NY99_ by using a fixed-cell ELISA (*11*). The mAbs and their characteristics follow: mAb 10C6, anti–nonstructural protein (NS) 1 (reactive with MVEV); mAb 10A1, anti-envelope glycoprotein (anti-E; specific for WNV_KUN_); mAb 2B2, anti-E (reactive with WNV); mAb 3.1112G, anti-NS1 (reactive with WNV); mAb 5H1, anti-NS5 (strong reaction with WNV_KUN_ strains, weak for WNV_NY99_, nonreactive with WNV strains from other lineages); mAb 17D7, anti-E (specific for strains of WNV with glycosylated E); mAb 3.101C, anti-E (specific for strains of WNV with unglycosylated E); and the pan–flavivirus-reactive mAbs (i.e., 4G4, anti-NS1; and 4G2, anti-E) (*12**–**15*; J. Hobson-Peters et al., unpub. data).

### Nucleic Acid Sequencing

For sequencing of the whole genome, we used total nucleic acid purified from virus-infected cell culture supernatant as template in 5 RT-PCRs with primers designed to cover the coding regions of any WNV genome (Table 1). RT and amplification were performed by using the SuperScript III One-Step RT-PCR System (Invitrogen, Carlsbad, CA, USA) with primers at 20 μM. RT was performed at 50°C for 30 min, followed by denaturation at 94°C for 2 min. PCR amplification involved 40 cycles (95°C for 30 s, 55°C for 1 min, 68°C for 4.5 min) followed by a final extension at 68°C for 10 min. Reaction products were visualized after electrophoresis on a ethidium bromide–stained 1% agarose gel. Reaction products were purified directly (MinElute PCR Purification Kit; QIAGEN, Valencia, CA, USA) or excised from the gel and cleaned (Gel MinElute PCR Purification Kit; QIAGEN). Purified nucleic acid was sequenced at the Australian Genome Research Facility (Sydney) by using the primers used to generate the PCR product. Each RT-PCR was run 3× and sequenced in both forward and reverse orientation. Sequence data were assembled by using Sequencher software (Gene Codes Corp., Ann Arbor, MI, USA). For subsequent isolates from horses and mosquitos, we used primers designed from the sequence of the WNV_NSW2011_ genome (Table 1) to amplify and sequence the NS3 and NS5 regions, in which changes had been identified. The same RT-PCR and sequencing methods were used, except that the annealing temperature was 50°C and extension time was 1 min. The nucleic acid sequences were translated and then aligned with WNV_NSW2011_ and WNV_KUN_ (GenBank accession nos. JN887352 and D00246.1, respectively) by using ClustalW (www.clustal.org).

**Table 1:** Primers used for viral RNA amplification and genomic sequencing of WNV isolates from horses and mosquitoes, Australia, 2011*

RT-PCR region	Forward primer, 5′ → 3′ (relative genome position†)	Reverse primer, 5′ → 3′ (relative genome position†)
Amplification and sequencing of whole genome		
5′ NTR capsid	TAGTTCGCCTGTGTGAGCTG (5′ NTR-2)	TTGAAAATTCCACAGGAATGG (capsid-1772)
Capsid-NS2A	GTGATAGCATTGGGCTCWCA (capsid-1720)	ATCTTGAAGGYYGCCATGAG (NS2A-1760)
NS2A-NS3	CACTGATGTGTTACGCTATGTCA (NS2A-3678)	CAAAGTCCCAATCATCGTTCT (NS3-5807)
NS3-NS5	CGGTTTGGTTTGTGCCTAGT (NS3-5687)	CCAACTTCACGCAGGATGTA (NS5-9235)
NS5–3′ NTR	GACCACTGGCTTGGAAGAAA (NS5-9169)	CTGGTTGTGCAGAGCAGAAG (3′ NTR-10955)
Partial sequencing of key regions of genome		
NS3	GTGCTGGTAAAACAAGGAGG (NS3-5201)	TGTATCCTCTAGCCGCGATG (NS3-5493)
NS5	TCGGCCCAGATGATGTG (NS5-9575)	CGGCATGGAACCACCAGTGTTC (NS5-9860)

*Primers were designed from available sequences in GenBank to cover the coding regions of any WNV genome. WNV, West Nile virus; RT, reverse transcription; NTR, nontranslated region; NS, nonstructural protein. †WNV_NY99_ GenBank accession no. NC_009942.1.

### Bioinformatics Analysis

Complete coding regions of selected WNV isolates, representing all lineages and clades and including all complete KUN sequences, were aligned with the WNV_NSW2011_ sequence as described (*16*). This alignment was transferred to BioEdit (www.mbio.ncsu.edu/BioEdit/bioedit.html) for manual editing before construction of phylogenetic trees. Maximum-likelihood trees were constructed by using PhyML (*17*). Trees were rooted by using the Japanese encephalitis virus Nakayama sequence (GenBank accession no. EF571853), which was removed from the final tree for clarity.

### Endoglycosidase Digestion

To examine glycosylation of the E protein, viral proteins from cultures of infected C6/36 cells were digested as described (*18*). Proteins were separated and analyzed by Western blot. Samples were loaded with reducing sodium dodecyl sulfate–polyacrylamide gel electrophoresis buffer (NuPAGE LDS Sample Buffer; Invitrogen) on a 4%–12% NuPAGE Gel (Invitrogen). Electrophoresed proteins were electroblotted onto nitrocellulose paper (Hybond C; GE Healthcare, Little Chalfont, UK) and immunostained with anti-E mAb (*11*).

### Plaque Morphology

We allowed the virus to adsorb to monolayers of Vero 76 cells in 6-well plates for 2 h at 37°C. The cells were overlaid with DMEM containing 0.75% low melting point agarose and 2% FBS. Four days after infection, the cells were fixed with 4% formaldehyde solution and stained with 0.2% crystal violet.

### Virulence in Mice

Groups of 10 weanling (18–19 days old) or young adult (4 weeks old) Swiss outbred CD1 mice were injected intraperitoneally with 10-fold dilutions of virus. The mice were monitored for 21 days after injection and euthanized when signs of encephalitis were evident. All animal procedures had received prior approval from The University of Queensland Animal Ethics Committee.

## Results

### Virus Isolation and Initial Characterization

Viral RNA was detected by WNV-specific rRT-PCR in fresh brain tissue from 6 of 12 horses showing signs of encephalitis. Viruses were isolated from 4 of these samples; each showed distinct cytopathology in BHK21 and Vero cells. rRT-PCR of the culture fluids and immunoperoxidase staining of the cells with pan–flavivirus-reactive and WNV-specific mAbs confirmed the isolation of a West Nile–like virus. The first isolate was named NSW2011 and designated WNV_NSW2011_. Eight isolates of WNV_NSW2011_ were isolated from *Culex annulirostris* Skuse mosquitoes during the 2011 vector season. Of the 8 isolates, 5 were from mosquitoes collected in the Riverina region of southwestern NSW (Hanwood, 4 isolates; Barren Box, 1 isolate); 2 were from the Murray region in southern NSW (Mathoura, 1 isolate; Moama, 1 isolate); and 1 was collected at Lower Portland in the outer western Sydney region of NSW. No other isolates of WNV were obtained.

### Antigenic Analysis of WNV_NSW2011_

To antigenically type WNV_NSW2011_ in a fixed-cell ELISA (*11*), we used a panel of mAbs previously shown to differentiate between strains of WNV_KUN_ and other WNVs (*11**,**13**–**15**,**19**,**20*). The recognition patterns showed that the WNV_NSW2011_ isolate most closely resembled Australian WNV_KUN_ strains; the WNV_KUN_-specific mAb 10A1 reacted strongly with prototype WNV_KUN_ and WNV_NSW2011_ but not with WNV_NY99_ (Table 2). However, the anti-NS5 mAb 5H1, which is also specific for WNV_KUN_ isolates from Australia (*15*), failed to react with WNV_NSW2011_ and WNV_NY99_, but it bound strongly to WNV_KUN_. The reaction patterns of mAbs 17D7 and 3.101C, which react specifically with glycosylated and unglycosylated WNV E antigens, respectively (*11**,**14*; J. Hobson-Peters et al., unpub. data), indicated that, unlike the E protein of WNV_KUN_, the E protein of WNV_NSW2011_ is glycosylated.

**Table 2:** Binding pattern of monoclonal antibodies to the viral antigens of 3 WNV strains in fixed-cell ELISA, Australia, 2011*

Virus	Monoclonal antibody, by specificity													
	Pan-flavivirus†			WNV group			WNV_KUN_			Unglycosylated WNV E protein		Glycosylated WNV E protein		MVEV
	4G4, anti-NS1	4G2, anti-E		2B2, anti-E	3.91D, anti-E		10A1, anti-E	5H1, anti-NS5						10C6, anti-NS1
										3.101C		17D7		
WNV_NSW2011_	+	+		+	+		+	−		−		+		−
WNV_KUN_†	+	+		+	+		+	+		+		−		−
WNV_NY99_‡	+	+		+	+		−	−		−		+		−

*WNV, West Nile virus; KUN, Kunjin; E, envelope; MVEV, Murray Valley encephalitis virus; NS, nonstructural protein; NS, nonstructural protein; NSW, New South Wales; + positive; –, negative; NY, New York. †Prototype WNV_KUN_ strain MRM-61C. ‡North American WNV strain.

To assess the level of antigenic crossreactivity between WNV_NSW2011_, WNV_KUN_, and WNV_NY99_, we assessed neutralization titers for homologous and heterologous viruses in immune serum samples from the following sources: horses infected during the 2011 outbreak, horses infected with WNV_KUN_ in the Northern Territory of Australia several years earlier, and horses infected with WNV in the United States. Convalescent-phase serum samples from WNV_NSW2011_-immune horses had neutralizing titers similar to those of the homologous virus (WNV_NSW2011_) and of WNV_KUN_ and WNV_NY99_ (Table 3). Serum samples from WNV_KUN_-immune horses from Northern Territory and from WNV-immune animals from the United States showed a similar pattern of cross-neutralization. However, serum samples from horses from Northern Territory and the United States showed slightly less neutralizing efficiency of WNV_NSW2011_; this was likely due to a higher dose (≈4-fold) of virus in the assay (Table 3). Overall, these results are consistent with those in our previous reports showing a high level of cross-neutralization between WNV_KUN_ and WNV_NY99_ strains (*21**,**22*). 

**Table 3:** Neutralizing titers of serum samples from WNV–infected horses against 3 WNV strains, Australia, 2011*

Horse serum samples	% Inhibition of CPE/growth†							
	WNV_NSW2011_, 100 infectious units			WNV_KUN_, 26 infectious units			WNV_NY99_, 32 infectious units	
	80‡	100§		80	100		80	100
Control¶								
1	<20	<20		<20	<20		<20	<20
2	<20	<20		<20	<20		<20	<20
3	<20	<20		<20	<20		<20	<20
4	<20	<20		<20	<20		<20	<20
5	<20	<20		<20	<20		<20	<20
NSW#								
04	640	320		1,280	1,280		640	320
06	320	160		640	640		1,280	160
08	320	320		1,280	1,280		640	640
28	320	320		640	640		320	320
36	320	160		640	640		320	640
NT**								
111473	**80**	**20**		**640**	**320**		**160**	**160**
104714	**320**	**80**		**640**	**640**		**640**	**640**
110910	80	40		160	160		160	160
98727	40	40		160	160		80	80
WNV††								
1	**160**	**40**		**640**	**40**		**320**	**320**
2	**320**	**160**		**1,280**	**640**		**160**	**160**
3	**80**	**160**		**320**	**320**		**640**	**640**
4	**40**	**20**		**160**	**160**		**320**	**320**
5	**320**	**80**		**1,280**	**320**		**640**	**640**
mAb 3.91D‡‡	>2,560	>2,560		>2,560	>2,560		>2,560	>2,560

*Determined, as described (*14*), by microneutralization assay in Vero cells. WNV, West Nile virus; CPE, cytopathic effect; NSW, New South Wales; KUN, Kunjin; NY, New York; NT, Northern Territory; mAb, monoclonal antibody. †**Boldface** indicates serum samples with >4-fold difference in titer between virus strains. ‡Determined by using a microscope to assess the level of CPE in each well compared with that in control wells. §Determined by the absence of viral antigen in the cell monolayer of each well when tested with a WNV-reactive mAb in ELISA. ¶Samples from uninfected horses. #Samples from horses infected with WNV during the 2011 outbreak in New South Wales, Australia. **Samples from horses infected with WNV_KUN_ in Northern Territory, Australia. ††Samples from horses infected with WNV in the United States. ‡‡This mAb has potent WNV-neutralizing activity (*11*).

### Nucleotide and Amino Acid Sequence Analysis of WNV_NSW2011_

**Figure 2:**
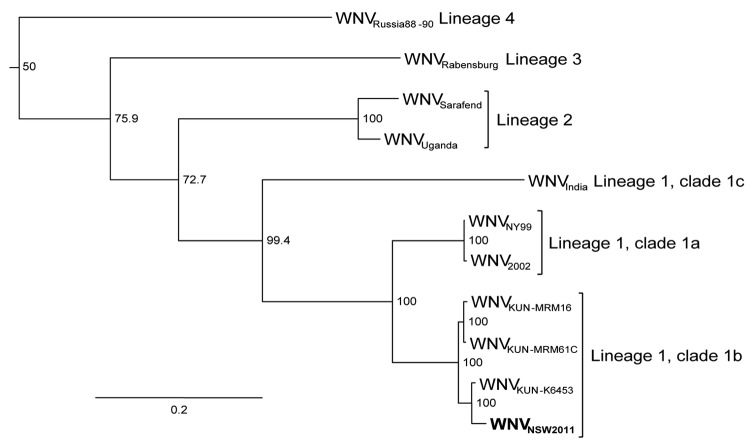
Maximum-likelihood tree based on nucleotide sequences of the complete open reading frame of genomes of West Nile virus (WNV) NSW2011 (**boldface**) and representative strains of WNV from the different lineages and clades. All published complete Kunjin (KUN) virus sequences are included. Bootstrap values are shown on the nodes and are expressed as a percentage of 1,000 replicates. Sequences downloaded from GenBank were WNV_Russia88–90_, AY277251; WNV_Rabensburg_, AY765264; WNV_Sarafend_, AY688948; WNV_Uganda_, AY532665; WNV_India_, DQ256376; WNV_NY99_, AF196835; WNV_2002_, GU827998; WNV_KUNV-MRM16_, GQ851602; WNV_KUNV-MRM61C_, AY274504; and WNV_KUNV-K6453_, GQ851603. NY, New York; NSW, New South Wales. Horizontal branch lengths indicate genetic distance proportional to the scale bar.

A comparison of the nucleotide sequence of the complete coding region of the first isolate of WNV_NSW2011_ with sequences available in GenBank confirmed that WNV_NSW2011_ was genetically most closely related to Australian WNV_KUN_ isolates (Figure 2). A detailed comparison of deduced amino acid sequences for the entire coding region of WNV_NSW2011_, WNV_KUN_, and WNV_NY99_ further confirmed a closer relationship between WNV_NSW2011_ and WNV_KUN_ than between WNV_NSW2011_ and WNV_NY99._ There was a 42-aa difference (18 nonconserved changes) between WNV_NSW2011_ and WNV_KUN_ and an 89-aa difference (38 nonconserved changes) between WNV_NSW2011_ and WNV_NY99_ (Table A1). At least 2 of the known WNV virulence markers present in WNV_NY99_ but not in WNV_KUN_ were found in WNV_NSW2011_ (Table A1). The glycosylation tripeptide (N-Y-S) at residues E_154–156_, which allows N-linked glycosylation at a conserved site on the E protein, domain I, and is associated with virulence in most WNV strains (*23*), was present in WNV_NSW2011_; its presence is consistent with the mAb recognition profile. A phenylalanine residue at aa 653 in NS5, which also is associated with enhanced virulence of WNV strains (*24*), was present in WNV_NSW2011_. Because the WNV_KUN_ strain–specific mAb 5H1 did not react with WNV_NSW2011_, we also examined the predicted amino acid sequence of the NS5 protein that corresponded to the linear epitope previously mapped for 5H1 to residues 41–53 in the methyltransferase domain (*15*). WNV_NSW2011_ and WNV_NY99_ contain a substitution (I→V) at residue 49 that is not contained in WNV_KUN_, confirming the critical role of this residue for 5H1 binding. Together these data suggest that WNV_NSW2011_ represents a virulent WNV strain that has emerged in Australia. However, the amino acid substitution in NS3, which is believed to be associated with increased virulence of WNV_NY99_ in birds in North America (*25*), was not present in isolate WNV_NSW2011_. Sequencing of the key regions of the other WNV isolates obtained during this outbreak (3 from horses and 8 from mosquitoes) showed that each was indistinguishable from WNV_NSW2011_.

### In vitro Growth Properties and E Protein Glycosylation Status of WNV_NSW2011_

**Figure 3:**
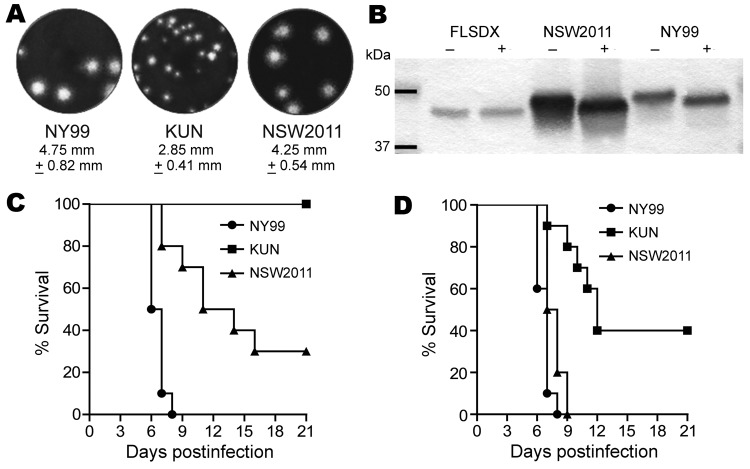
Studies of West Nile virus (WNV) properties in cell cultures and mice. A) Plaque morphology of WNV_NY99_, prototype WNV_KUN,_ and WNV_NSW2011_ in Vero cells. Cells in 6-well plates were infected with specified virus and overlaid with 0.75% low melting point agarose in Dulbecco modified minimum essential medium (Life Technologies, Carlsbad, CA, USA) containing 2% fetal bovine serum. Four days after infection, the cells were fixed with 4% formaldehyde and stained with 0.2% crystal violet. B) Assessment of envelope (E) protein glycosylation of WNV_NSW2011_, WNV_KUN_ and WNV_NY99_ by endoglycosidase digestion (PNGase F; Roche Diagnostics, Basel, Switzerland). Viral proteins in culture supernatant were digested by PNGase F (+) or undigested (−) and then resolved on sodium dodecyl sulfate–polyacrylamide gel electrophoresis. The migration rate of the E protein in each sample was determined by Western blot with E glycoprotein–specific monoclonal antibodies. C) Young adult (4 weeks old) or D) weanling (18–19 days old) Swiss outbred mice survival after intraperitoneal injection with 1,000 PFU (adult) or 10 PFU (weanling) of WNV_NY99_, WNV_KUN_, or WNV_NSW2011_. The mice were monitored for 21 days after injection for signs of encephalitis and then euthanized. The differences in virulence in weanling and adult mice between different pairs of viruses were all highly significant, as calculated by log rank Mantel-Cox algorithm with exact p values: for adult mice, WNV_NY99_ vs. WNV_KUN_ p<0.0001, WNV_NY99_ vs. WNV_NSW2011_ p = 0.0001, and WNV_KUN_ vs. WNV_NSW2011_ p = 0.0012; and for weanling mice, WNV_NY99_ vs. WNV_KUN_ p<0.0001, WNV_NY99_ vs. WNV_NSW2011_ p =0.0004, and WNV_KUN_ vs. WNV_NSW2011_ p = 0.0006. NY, New York; KUN, Kunjin; NSW, New South Wales.

The average plaque size of WNV_NSW2011_ (Figure 3, panel A) was 4.2 mm ± 0.5 mm, much closer to WNV_NY99_ (4.7 mm ± 0.8 mm) than to the prototype WNV_KUN_ (2.8 mm ± 0.4 mm). To confirm the presence of an N-linked glycan on the E protein of WNV_NSW2011_, we used Western blot to analyze endoglycosidase-digested viral protein. Analysis showed that the E protein of WNV_NSW2011_ and WNV_NY99_ migrated slightly faster than the undigested control protein. This result is consistent with N-linked glycosylation (Figure 3, panel B). However, consistent with the lack of a potential glycosylation site on the E protein of most WNV_KUN_ isolates, we found no evidence of N-linked glycosylation for WNV_KUN_ (*11**,**20**,**26*).

### Neuroinvasive Properties of WNV_NSW2011_ in mice

Injection of 18- to 19-day-old (weanling) mice with 10-fold dilutions of virus showed that substantially lower doses of WNV_NSW2011_ (50% lethal dose [LD_50_] 0.5 PFU), compared with WNV_KUN_ (LD_50_ 13.4 PFU), induced neurologic signs (Table 4); and the time to disease onset was substantially shorter (Table 4; Figure 3, panel C). In contrast, the LD_50_ for WNV_NY99_ (0.1 PFU) was lower than that for WNV_NSW2011_, and neurologic signs developed more rapidly (Table 4; Figure 3, panel C). Only WNV_NY99_ and WNV_NSW2011_ caused a substantial number of deaths among 4-week-old (young adult) mice. Compared with WNV_NSW2011_, WNV_NY99_ exhibited a lower LD_50_ (240 PFU vs. 0.7 PFU, respectively) and a shorter time to death (10.7 days vs. 6.6 days, respectively, at 1,000 PFU) (Table 5; Figure 3, panel C).

**Table 4:** Virulence of 3 WNVs in 18- to19-day-old mice after intraperitoneal injection, Australia, 2011*

Virus and dose, PFU	No. mice/no. died	Average survival time, d	LD_50_
WNV_NY99_			
100	10/10	6.1	
10	10/10	6.7	
1	10/10	6.9	0.1 PFU
0.1	5/10	7.8	
WNV_KUN_			
1,000	9/10	8.4	
100	4/10	8	13.4 PFU
10	6/10	10.2	
1	3/10	12	
WNV_NSW2011_			
1,000	10/10	7.1	
100	10/10	7.4	
10	10/10	7.7	0.5 PFU
1	7/10	8.3	
0.1	1/10	10	

*WNV, West Nile virus; LD_50_, dose at which 50% of the mice died; NY, New York; KUN, Kunjin; NSW, New South Wales.

**Table 5:** Virulence of 3 WNVs in 4-week-old mice after intraperitoneal injection, Australia, 2011*

Virus and dose, PFU	No. mice/no. died	Average survival time, d	LD_50_
WNV_NY99_			
100	10/10	8.3	
10	10/10	8.2	0.7 PFU
1	5/10	8.6	
0.1	2/10	10	
WNV_KUN_			
1,000	0/10	21	>1,000 PFU
WNV_NSW2011_			
1,000	7/10	10.7	
100	2/10	11	240 PFU
10	3/10	10.3	

*WNV, West Nile virus; LD_50_, dose at which 50% of the mice died; NY, New York; KUN, Kunjin; NSW, New South Wales.

## Discussion

It is estimated that at least 1,000 horses were affected during an unprecedented outbreak of encephalitis in southeastern Australia during 2011. The case-fatality rate was 10%–15%, and diseased animals had clinical signs consistent with those observed during a WNV outbreak in the United States. Not only was the Australian outbreak unique and unprecedented in size and disease severity, but its epidemiologic features also differed from those observed previously in Australia. In particular, WNV_KUN_ now has been detected on the eastern seaboard of NSW, close to major urban areas, including the largest 3 cities (Sydney, Newcastle, and Wollongong). This detection occurred despite relatively small mosquito populations in many of these areas, suggesting that the virus is more virulent and probably transmitted more efficiently than other strains between mosquito vectors and mammalian hosts. Characterization of virus isolated from the brain of an animal that died showed a variant strain of WNV most closely related to WNV_KUN_. Typing of WNV_NSW2011_ by reactivity with a panel of mAbs indicated the virus was antigenically more similar to the native Australian WNV_KUN_ strains than to exotic WNV strains. However, for WNV_NSW2011_, the reaction profile of mAbs 17D7 and 3.101C differed from that of the prototype WNV_KUN_. Similar to WNV_NY99_ and other virulent strains of WNV, WNV_NSW2011_ E protein was glycosylated at residue 154. This finding was further confirmed by gene sequencing and endoglycosidase F digestion analysis. Glycosylation of WNV E protein at this site is thought to enhance virus dissemination in the infected host by increasing the efficiency of assembly and release of virus particles from infected cells (*27*). Previous studies showed that a phenylalanine residue at aa 653 in NS5, observed in WNV_NSW2011_ and WNV_NY99_ but not in WNV_KUN_, is associated with increased resistance to interferon, which may also enhance virulence in the host (*24*). Virulence studies in weanling and young adult mice clearly demonstrate that WNV_NSW2011_ is substantially more neuroinvasive than the prototype strain of WNV_KUN_, which might explain the severity of the 2011 outbreak. However, the association between the identified and perhaps other amino acid changes and increased virulence of WNV_NSW2011_ in horses and mice will require further confirmation by using site-directed mutagenesis of an infectious cDNA clone.

Another unusual aspect of the 2011 outbreak was the absence of encephalitis caused by WNV_KUN_ in humans. In contrast, several confirmed cases of Murray Valley encephalitis in humans were recorded in southeastern Australia during this time. This absence of disease in humans suggests that ecologic and/or epidemiologic features of the virus transmission cycle, such as small mosquito populations and timely alerts, probably resulted in less exposure of the human population to WNV_NSW2011_.

The US outbreak of WNV was associated with high mortality among several bird species, particularly American crows (*Corvus brachyrhynchos*). In contrast, increased mortality among birds of any species was not reported during the 2011 outbreak in southeastern Australia. The lack of disease in birds in Australia supports the hypothesis that the amino acid substitution observed in WNV_NY99_ (Ala→Pro at aa 249 in NS3) (*25*) is associated with increased virulence in birds because this change was not present in the WNV_NSW2011_ isolate. However, this observation should be viewed with some caution because of the species differences between birds in Australia and the United States and because disease was limited or absent when a species of Australian crow (Little Raven [*Corvus mellori*]) was experimentally infected with WNV_NY99_ (*28*).

Taken together, our results show that the WNV_NSW2011_ isolate is closely related to Australian WNV_KUN_ strains. However, in contrast to the prototype WNV_KUN_ strain (MRM-61C), the new virus has several amino acid substitutions that are likely to be the reason for enhanced virulence in horses. More extensive epidemiologic studies in the field and experimental studies in the laboratory are required to determine the relation of WNV_NSW2011_ to other currently and previously circulating WNV_KUN_ strains and to confirm which viral proteins and amino acid residues are associated with increased virulence of WNV_NSW2011_ in horses.

## Appendix

**Table A1:** Amino acid differences between WNV prototype Kunjin MRM61C, NY99, and NSW11 strains*

Polyprotein and aa position in polyprotein (position in individual protein)	WNV strain (GenBank accession no.)			No. aa differences†
	MRM61C AY274504.1)	NY99 4132 (HQ596519)	NSW2011 (JN887352)	
C				
28	T	I	I	
41	R	K	R	2(0):2(2):4(2)
44	T	I	I	
71	S	G	S	
PrM				
108(3)	K	K	R	
113(8)	F	V	F	
114(9)	M	M	T	
120(15)	G	S	G	
143(38)	T	T	A	6(5):10(7):7(3)
145(40)	I	V	I	
158(53)	I	I	T	
166(61)	H	Y	Y	
195(90)	L	S	L	
228(123)	S	A	S	
279(174)	A	V	T	
E				
334(44)	K	K	R	
357(67)	E	D	E	
379(89)	S	A	S	
383(93)	K	R	K	
416(126)	T	I	T	
446(156)	F	**S**	**S**	
449(159)	T	V	T	3(1):15(7):14(8)
452(162)	A	T	A	
489(199)	S	N	S	
519(229)	E	G	E	
521(231)	N	T	N	
600(310)	R	K	R	
628(338)	I	V	I	
655(365)	S	A	S	
700(410)	A	T	A	
773(483)	L	L	F	
NS1				
837(46)	I	I	V	
879(88)	I	V	I	
893(102)	R	K	R	
926(135)	I	V	I	
937(146)	Q	Q	R	
961(170)	R	K	R	
997(206)	F	L	L	5(2):12(2):9(0)
1027(236)	V	I	V	
1036(245)	I	V	I	
1055(264)	S	N	S	
1081(290)	S	S	N	
1089(298)	T	T	I	
1118(327)	N	S	N	
NS2A				
1255(112)	A	V	V	
1262(119)	Y	H	Y	
1272(129)	M	I	I	
1311(168)	C	R	C	5(0):5(2):6(2)
1326(183)	I	I	V	
1330(187)	I	I	M	
1355(212)	F	L	F	
1366(223)	V	I	I	
NS2B				
1400(26)	I	I	M	
1438(64)	G	S	S	2(1):2(0):2(1)
1477(103)	A	V	A	
1520(15)	R	K	K	
1615(110)	Q	R	Q	
1680(174)	V	I	V	
1754(249)	A	P	A	
1809(303)	R	K	R	
1836(331)	A	S	A	3(0):12(6):11(6)
1861(356)	I	T	I	
1887(382)	K	K	R	
1889(383)	I	V	I	
1912(406)	V	I	V	
1970(465)	N	N	S	
1991(486)	C	F	C	
2115(610)	S	A	S	
NS4A				
2129(5)	F	L	F	
2179(55)	A	A	T	
2209(85)	V	A	V	
2213(89)	A	V	A	2(2):5(1):5(1)
2265(141)	L	M	L	
2269(145)	G	S	S	
NS4B				
2288(15)	G	S	G	
2296(23)	T	V	I	
2302(29)	I	M	I	
2324(50)	V	V	F	
2334(60)	T	T	M	7(4):11(5):7(3)
2368(94)	A	A	S	
2387(114)	S	A	S	
2389(116)	T	T	A	
2449(176)	V	I	V	
2450(177)	M	M	I	
2459(186)	L	V	L	
2518(245)	V	I	I	
NS5				
2553(25)	I	T	I	
2561(33)	T	I	T	
2575(47)	R	G	R	
2577(48)	I	V	V	
2629(101)	R	R	K	
2690(162)	L	I	L	
2705(177)	K	R	K	7(3):15(7):12(8)
2711(183)	V	V	I	
2775(247)	K	R	K	
2840(311)	E	D	E	
3059(531)	R	K	R	
3088(560)	D	D	N	
3181(653)	S	**F**	**F**	
3247(719)	T	T	I	
3259(731)	T	V	T	
3405(877)	S	A	S	
3427(899)	L	L	F	
Total changes				42(18):89(38):77(34

*WNV, West Nile virus; NY, New York; NSW, New South Wales; C, core; NS, nonstructural; PrM, precursor membrane; E, envelope. †No. aa differences (nonconservative changes in parentheses) for WNV_KUN_ vs. WNV_NSW2011_:WNV_NY99_ vs. WNV_NSW2011_:WNV_KUN_ vs. WNV_NY99_.

## References

[1] Russell RC, Dwyer DE. Arboviruses associated with human disease in Australia. Microbes Infect. 2000;2:1693–704. 10.1016/S1286-4579(00)01324-1 11137043

[2] Mackenzie JS, Lindsay MD, Coelen RJ, Broom AK, Hall RA, Smith DW. Arboviruses causing human disease in the Australasian zoogeographic region. Arch Virol. 1994;136:447–67. 10.1007/BF01321074 8031248

[3] Hall RA, Broom AK, Smith DW, Mackenzie JS. The ecology and epidemiology of Kunjin virus. Curr Top Microbiol Immunol. 2002;267:253–69. 10.1007/978-3-642-59403-8_13 12082993

[4] Badman RT, Campbell J, Aldred J. Arbovirus infection in horses—Victoria 1984. Commun Dis Intell. 1984;17:5–6.

[5] Ostlund EN, Crom RL, Pedersen DD, Johnson DJ, Williams WO, Schmitt BJ. Equine West Nile encephalitis, United States. Emerg Infect Dis. 2001;7:665–9. 10.3201/eid0704.010412 11589171 PMC2631754

[6] Russell RC, Doggett SL, Clancy J, Haniotis J, Patsouris K, Hueston L, Arbovirus and vector surveillance in NSW, 1997–2000. Arbovirus Research in Australia. 2001;8:304–13.

[7] Khromykh AA, Sedlak PL, Westaway EG. Complementation analysis of the flavivirus Kunjin NS5 gene reveals an essential role for translation of its N-terminal half in RNA replication. J Virol. 1999;73:9247–55. 10516033 10.1128/jvi.73.11.9247-9255.1999 PMC112959

[8] Audsley M, Edmonds J, Liu W, Mokhonov V, Mokhonova E, Melian EB, Virulence determinants between New York 99 and Kunjin strains of West Nile virus. Virology. 2011;414:63–73. 10.1016/j.virol.2011.03.008 21477835 PMC3089702

[9] Hall RA, Broom AK, Hartnett AC, Howard MJ, Mackenzie JS. Immunodominant epitopes on the NS1 protein of MVE and KUN viruses serve as targets for a blocking ELISA to detect virus-specific antibodies in sentinel animal serum. J Virol Methods. 1995;51:201–10. 10.1016/0166-0934(94)00105-P 7738140

[10] Eiden M, Vina-Rodriguez A, Hoffmann B, Ziegler U, Groschup M. Two new real-time quantitative reverse transcription polymerase chain reaction assays with unique target sites for the specific and sensitive detection of lineages 1 and 2 West Nile virus strains. J Vet Diagn Invest. 2010;22:748–53. 10.1177/104063871002200515 20807934

[11] Adams SC, Broom AK, Sammels LM, Hartnett AC, Howard MJ, Coelen RJ, Glycosylation and antigenic variation among Kunjin virus isolates. Virology. 1995;206:49–56. 10.1016/S0042-6822(95)80018-2 7530394

[12] Hall RA, Kay BH, Burgess GW, Clancy P, Fanning ID. Epitope analysis of the envelope and non-structural glycoproteins of Murray Valley encephalitis virus. J Gen Virol. 1990;71:2923–30. 10.1099/0022-1317-71-12-2923 1703213

[13] Hall RA, Burgess GW, Kay BH, Clancy P. Monoclonal antibodies to Kunjin and Kokobera viruses. Immunol Cell Biol. 1991;69:47–9. 10.1038/icb.1991.7 1651286

[14] Hobson-Peters J, Toye P, Sanchez MD, Bossart KN, Wang LF, Clark DC, A glycosylated peptide in the West Nile virus envelope protein is immunogenic during equine infection. J Gen Virol. 2008;89:3063–72. 10.1099/vir.0.2008/003731-0 19008394

[15] Hall RA, Tan SE, Selisko B, Slade R, Hobson-Peters J, Canard B, Monoclonal antibodies to the West Nile virus NS5 protein map to linear and conformational epitopes in the methyltransferase and polymerase domains. J Gen Virol. 2009;90:2912–22. 10.1099/vir.0.013805-0 19710254

[16] May FJ, Davis CT, Tesh RB, Barrett AD. Phylogeography of West Nile virus: from the cradle of evolution in Africa to Eurasia, Australia, and the Americas. J Virol. 2011;85:2964–74. 10.1128/JVI.01963-10 21159871 PMC3067944

[17] Guindon S, Gascuel O. A simple, fast, and accurate algorithm to estimate large phylogenies by maximum likelihood. Syst Biol. 2003;52:696–704. 10.1080/10635150390235520 14530136

[18] Prow NA, May FJ, Westlake DJ, Hurrelbrink RJ, Biron RM, Leung JY, Determinants of attenuation in the envelope protein of the flavivirus Alfuy. J Gen Virol. 2011;92:2286–96. 10.1099/vir.0.034793-0 21733886

[19] Lanciotti RS, Roehrig JT, Deubel V, Smith J, Parker M, Steele K, Origin of the West Nile virus responsible for an outbreak of encephalitis in the northeastern United States. Science. 1999;286:2333–7. 10.1126/science.286.5448.2333 10600742

[20] Scherret JH, Poidinger M, Mackenzie JS, Broom AK, Deubel V, Lipkin WI, The relationships between West Nile and Kunjin viruses. Emerg Infect Dis. 2001;7:697–705. 10.3201/eid0704.010418 11585535 PMC2631745

[21] Hall RA, Nisbet DJ, Pham KB, Pyke AT, Smith GA, Khromykh AA. DNA vaccine coding for the full-length infectious Kunjin virus RNA protects mice against the New York strain of West Nile virus. Proc Natl Acad Sci U S A. 2003;100:10460–4. 10.1073/pnas.1834270100 12917491 PMC193583

[22] Chang DC, Liu WJ, Anraku I, Clark DC, Pollitt CC, Suhrbier A, Single-round infectious particles enhance immunogenicity of a DNA vaccine against West Nile virus. Nat Biotechnol. 2008;26:571–7. 10.1038/nbt1400 18425125

[23] Beasley DW, Li L, Suderman MT, Barrett AD. Mouse neuroinvasive phenotype of West Nile virus strains varies depending upon virus genotype. Virology. 2002;296:17–23. 10.1006/viro.2002.1372 12036314

[24] Laurent-Rolle M, Boer EF, Lubick KJ, Wolfinbarger JB, Carmody AB, Rockx B, The NS5 protein of the virulent West Nile virus NY99 strain is a potent antagonist of type I interferon-mediated JAK-STAT signaling. J Virol. 2010;84:3503–15. 10.1128/JVI.01161-09 20106931 PMC2838099

[25] Brault AC, Huang CY, Langevin SA, Kinney RM, Bowen RA, Ramey WN, A single positively selected West Nile viral mutation confers increased virogenesis in American crows. Nat Genet. 2007;39:1162–6. 10.1038/ng2097 17694056 PMC2291521

[26] Wright PJ. Envelope protein of the flavivirus Kunjin is apparently not glycosylated. J Gen Virol. 1982;59:29–38. 10.1099/0022-1317-59-1-29 6279774

[27] Hanna SL, Pierson TC, Sanchez MD, Ahmed AA, Murtadha MM, Doms RW. N-linked glycosylation of West Nile virus envelope proteins influences particle assembly and infectivity. J Virol. 2005;79:13262–74. 10.1128/JVI.79.21.13262-13274.2005 16227249 PMC1262570

[28] Bingham J, Lunt RA, Green DJ, Davies KR, Stevens V, Wong FY. Experimental studies of the role of the little raven (*Corvus mellori*) in surveillance for West Nile virus in Australia. Aust Vet J. 2010;88:204–10. 10.1111/j.1751-0813.2010.00582.x 20553567

